# Early development of olfactory circuit function

**DOI:** 10.3389/fncel.2023.1225186

**Published:** 2023-07-26

**Authors:** Joost X. Maier, Zihao Zhang

**Affiliations:** Department of Neurobiology and Anatomy, Wake Forest School of Medicine, Winston-Salem, NC, United States

**Keywords:** piriform cortex, olfactory bulb, neonatal, local field potential, oscillation, inhibition

## Abstract

During early development, brains undergo profound changes in structure at the molecular, synaptic, cellular and circuit level. At the same time, brains need to perform adaptive function. How do structurally immature brains process information? How do brains perform stable and reliable function despite massive changes in structure? The rodent olfactory system presents an ideal model for approaching these poorly understood questions. Rodents are born deaf and blind, and rely completely on their sense of smell to acquire resources essential for survival during the first 2 weeks of life, such as food and warmth. Here, we review decades of work mapping structural changes in olfactory circuits during early development, as well as more recent studies performing *in vivo* electrophysiological recordings to characterize functional activity patterns generated by these circuits. The findings demonstrate that neonatal olfactory processing relies on an interacting network of brain areas including the olfactory bulb and piriform cortex. Circuits in these brain regions exhibit varying degrees of structural maturity in neonatal animals. However, despite substantial ongoing structural maturation of circuit elements, the neonatal olfactory system produces dynamic network-level activity patterns that are highly stable over protracted periods during development. We discuss how these findings inform future work aimed at elucidating the circuit-level mechanisms underlying information processing in the neonatal olfactory system, how they support unique neonatal behaviors, and how they transition between developmental stages.

## Introduction

Brains undergo extensive structural changes on multiple time scales throughout life. This is particularly apparent during early development when neurons migrate, differentiate, change morphology, and form balanced excitatory and inhibitory connections (Tau and Peterson, 2010; Sarma et al., 2011). Eventually, this process establishes mature processing circuits that are the subject of study for the bulk of neuroscience research. However, long before reaching their adult state, brains are highly functional and capable of mediating adaptive behaviors in some domains. Thus, despite structural immaturity at the cellular, molecular and synaptic level, “immature” fails to capture certain functional capabilities of the developing brain. Moreover, whereas the extant literature has focused extensively on how brain structure develops from immature to mature, our understanding of how developing brains process information to mediate functional interactions with the environment *in vivo* is severely lagging (Avitan and Goodhill, 2018). We argue that the olfactory system presents an ideal model for studying information processing in the developing brain. In many species, sensory systems like vision and audition undergo postnatal development largely uncoupled from adaptive behavioral output (Mooney et al., 1996; Hanganu et al., 2006; Colonnese et al., 2010). For example, rodents—a major model system for studying the neural basis of sensory processing and behavior—remain deaf and blind during the first 2 weeks of life. Proper early life development of sensory processing capabilities in the visual and auditory systems relies on internally-generated spontaneous activity patterns (i.e., retinal and cochlear waves, respectively) (Katz and Shatz, 1996; Mooney et al., 1996; Weliky and Katz, 1999; Tritsch et al., 2007, 2010). The contribution of natural sensory input to circuit development in the visual system finds its onset after the second week of life, during the so-called critical period (Crair et al., 1998; Rochefort et al., 2009; Hoy and Niell, 2015; Hooks and Chen, 2020). In the olfactory system, early development and maintenance of proper connectivity also relies in part on spontaneously-generated activity patterns [(Yu et al., 2004; Lorenzon et al., 2015; Nakashima et al., 2019), reviewed in Redolfi and Lodovichi (2021)], suggesting a shared principle. However, unlike vision and audition, processing of external sensory input to the olfactory system is crucial for survival already at birth. Odor signals support the formation of bonds between pups and their caregivers, and guide behaviors that help pups acquire essential resources such as food and warmth (Landers and Sullivan, 2012).

Here, we review research on the structure and function of the neonatal olfactory system in rodents. Early work established the importance of olfaction for neonatal behavior and identified the brain regions involved in neonatal odor processing. Ongoing work at the molecular, cellular and synaptic level provides further insight into the local circuit motifs that characterize the neonatal olfactory network. Finally, recent studies started to characterize how these circuits interact to generate dynamic activity patterns that reflect sensory processing *in vivo*. The highlighted findings reveal the challenges of performing functional sensory processing in the structurally immature brain, and shed light on the strategies the brain has evolved to resolve these challenges at multiple levels of organization: from molecular to systems.

## Unique odor-guided behavior in neonates

A long history of elegant behavioral work in rats illustrates the crucial role of olfaction for survival of neonatal rodents. The ability to learn about the olfactory environment starts already at prenatal stages. Rats learn preferences for both natural and artificial odorants experienced *via* the amniotic fluid and in the first hours after birth (Pedersen and Blass, 1982; Miller and Spear, 2008, 2009). Similar patterns of prenatal olfactory learning have been observed in human infants (Schaal et al., 2020). At the time of birth, rats are capable of using prenatally exposed odors to guide suckling behavior (Leon et al., 1977; Alberts and Brunjes, 1978; Galef and Kaner, 1980; Pedersen and Blass, 1982; Rosenblatt, 1983; Leon, 1992), and show increased head movements in the context of exposed odors (Miller and Spear, 2008). The importance of olfaction for survival during this early developmental period is underscored by work on mice rendered anosmic through transgenics. Anosmia drastically decreases pups’ survival chances in the first couple of days after birth as a result of impaired suckling behavior (Brunet et al., 1996; Contos et al., 2000). Unique olfactory learning and behavior continues during the first 2 weeks of life, when rats are completely dependent on their mother for survival. Pups learn preferences for arbitrary odors associated with maternal care, or signals that mimic maternal care (warmth, tactile stimulation, milk infusion), after only 10 min of exposure (Caza and Spear, 1984; Sullivan and Leon, 1987; Kraebel and Spear, 2000; Miller and Spear, 2009). Subsequent exposure to the same odor evoked orienting and approach behavior. Thus, behavioral work suggests a highly functional sense of smell in neonatal rodents—one that is uniquely adapted to the needs of the animal at the earliest postnatal developmental stages.

## The brain network supporting olfaction during early life

**FIGURE 1:**
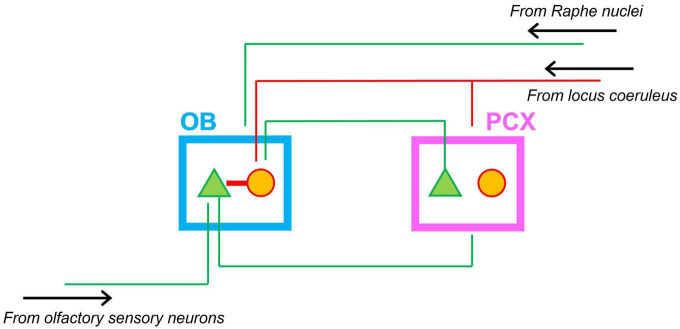
Schematic of the neonatal olfactory circuit. Afferent input from olfactory sensory neurons in the nasal epithelium targets mitral cells in the olfactory bulb (OB). Mitral cells in turn project to the piriform olfactory cortex (PCX). Feedback from the PCX targets granule cells in the OB. Granule cells in turn inhibit mitral cells in the OB. Neonatal interneurons in the PCX do not form functional inhibitory synapses. Both OB and PCX receive modulatory noradrenergic input from the locus coeruleus. Green triangles indicate excitatory projection neurons; red circles indicate interneurons. Green lines indicate excitatory projections; red lines indicate inhibitory projections. The OB also receives excitatory inputs from the Raphe nuclei (not shown).

To support neonatal odor-guided behaviors, a functional olfactory system emerges early in development. First order neurons in the olfactory epithelium (olfactory sensory neurons) innervate the olfactory bulb (OB) in anatomically segregated clusters called glomeruli and form functional synapses with mitral cells (the principal output neurons of the OB) prenatally (Hinds and Hinds, 1972; Mair and Gesteland, 1982; Malun and Brunjes, 1996; Treloar et al., 1999; Walz et al., 2006; Illig, 2007). The axons of mitral cells form the lateral olfactory tract (LOT) and innervate the piriform cortex by the time of birth (Schwob and Price, 1984; Walz et al., 2006). Thus, a primary olfactory circuit consisting of the OB and PCX that processes odor-driven input is established at the time of birth (Schwob et al., 1984; Illig, 2007; Zhang et al., 2020). Modulatory input to the OB-PCX circuit comes from several brainstem nuclei, including the locus coeruleus (LC) and Raphe nuclei, which release the neuromodulators norepinephrine (NE) and serotonin (5-HT), respectively. Figure 1 shows a schematic of the neonatal olfactory circuit and its main bottom-up and top-down input sources.

All regions in the network depicted in Figure 1 are involved in neonatal olfaction. Exposure to an artificial odor in the context of (signals associated with) maternal care produces selective changes in responsiveness of both OB (Coopersmith and Leon, 1984; Wilson et al., 1985, 1987; Wilson and Leon, 1988) and PCX (Roth and Sullivan, 2005; Morrison et al., 2013; Mukherjee et al., 2014) neurons, as measured by metabolic markers, *in vitro* recordings, and single unit recordings in anesthetized animals. Modulatory systems play an important role in driving plastic changes in the olfactory circuit. Blocking NE receptors in the OB (Sullivan et al., 1992) impairs neonatal preference learning, and so does lesioning the LC (Sullivan et al., 1994). Depletion of 5-HT in the OB has a comparable effect on odor preference learning (McLean et al., 1993). Moreover, pairing an odor stimulus with direct stimulation of the LC is sufficient to instill preferences for the paired odor (Sullivan et al., 2000). Similarly, infusion of the adrenergic antagonist propranolol to the PCX impairs preference learning for an odor stimulus in the context of signals mimicking maternal care; infusion of the adrenergic agonist isoproterenol induces a preference for a concurrent odor stimulus in the absence of signals mimicking maternal care.

## Circuit-level mechanisms underlying neonatal olfactory processing

Although circuit-level function in the neonatal olfactory system remains poorly understood, the extant literature suggests a set of unique sensory processing mechanisms that rely on a combination of mature and immature circuit motifs. Much of the work on the mechanisms underlying neonatal olfactory processing has focused on cellular, molecular and synaptic characteristics of circuit motifs locally within the OB and PCX.

The neonatal OB exhibits some remarkably mature features during the first week of postnatal life, most notably the presence of functional inhibitory synapses (Wilson and Leon, 1986; Wang et al., 2005; Mack-Bucher et al., 2007; Dietz et al., 2011). Early anatomical studies noted the presence of granule cells—the main inhibitory cell type in the OB (Hinds, 1968; Mair et al., 1982)—at birth. However, the vast majority of granule cells are of postnatal origin [∼90%, (Rosselli-Austin and Altman, 1979)], and numbers steadily increase until the third week of life (Hinds, 1968). Despite the low numbers of granule cells, subsequent work employing extracellular recordings in the OB of anesthetized neonatal rats showed that mitral cells show a phase of strong inhibition in response to antidromic electrical stimulation of the LOT (Wilson and Leon, 1986, 1987), and suggested that this inhibition is mediated *via* reciprocal mitral cell-granule cell synapses. This idea was supported by recent molecular and *in vitro* work demonstrating that GABA-mediated inhibition develops more quickly in the OB compared to the rest of the brain (Wang et al., 2005; Mack-Bucher et al., 2007; Dietz et al., 2011). Due to low expression levels of the potassium chloride co-transporter KCC2 in neonatal neurons, intracellular chloride concentrations are generally high, and GABA has a depolarizing, excitatory effect on postsynaptic cells. This phenomenon is widely observed in the neocortex and hippocampus of rats up to around P8 (Ben-Ari et al., 1989; Cherubini et al., 1991). In contrast, KCC2 levels in the neonatal OB are closer to adult levels, allowing for functional inhibition in the circuit (Wang et al., 2005).

In adults, granule cell-mediated inhibition plays a key role on in shaping odor representations in the OB through lateral inhibition (Schoppa and Urban, 2003). Granule cells are also the main recipient of centrifugal and modulatory projections in the adult (Price and Powell, 1970), which have been shown to affect odor discriminability (Otazu et al., 2015). In neonatal animals, feedback projections from the PCX (Schwob and Price, 1984) and LC (McLean and Shipley, 1991) mainly target the granule cell layer. Findings regarding the effect of adrenergic modulators on mitral cell excitability are consistent with the idea that modulatory inputs to the OB target inhibitory granule cells, thereby modulating OB output. It is currently unclear how inhibition in the neonatal OB affects odor representations. Future behavioral work will characterize to what extent neonatal animals are able to discriminate and/or generalize between odors, and what the role of cortical feedback and inhibition is. The observation that inhibition in the neonatal OB is stronger than in the adult suggests a unique adaptation to the neonatal state: enabling fast and robust learning of maternal odors by creating highly specific odor representations. Mechanistically, enhanced inhibition may be accomplished by dense feedback projections, since the number of granule cells is low in neonates.

Compared to the neonatal OB, inhibition in the PCX remains immature until the third week of life. In line with developmental dynamics previously observed in the neocortex and hippocampus, GABA has a depolarizing effect on postsynaptic PCX neurons until at least P8 (Pardo et al., 2018). Despite the lack of inhibition, PCX plays an important role in mediating neonatal odor preference learning, as reviewed above. *In vitro* work suggests a unique excitatory mechanism underlying experience-dependent plasticity in the PCX. Prior to maturation of the local piriform cortical circuit, feedforward OB→PCX synapses are highly plastic and rely on NMDA receptors (Franks and Isaacson, 2005). After the second week of life, the strength of NMDA-mediated OB→PCX synapse plasticity declines and makes way for mature intra-cortical plasticity involving AMPA receptors (Poo and Isaacson, 2007) and balanced excitatory/inhibitory synapses (Canto-Bustos et al., 2022).

## Dynamic activity patterns in the neonatal olfactory system *in vivo*

**FIGURE 2:**
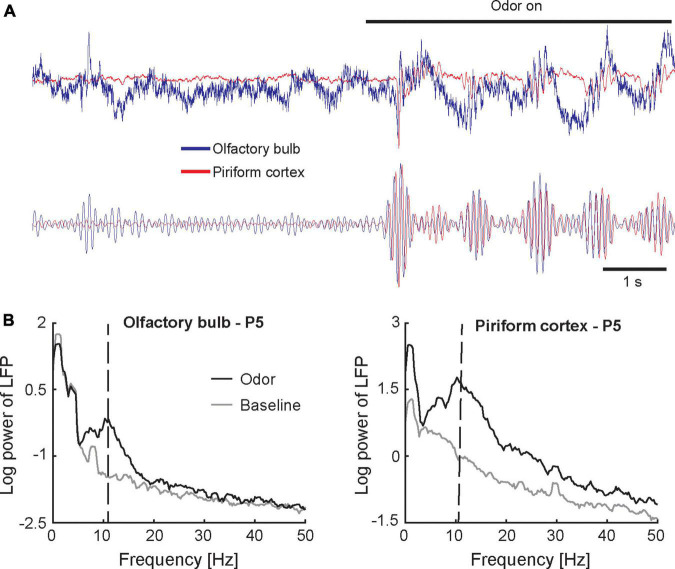
Oscillatory activity in the neonatal olfactory network. **(A)** Traces represent the raw (upper panel) and band-pass filtered (lower panel) local field potential recorded simultaneously from the OB (blue) and PCX (red) in a 5-day old awake rat pup (single trial). Inhaling an odor stimulus (amyl acetate; presence of odor stimulus is indicated by the horizontal black bar) elicits a burst of oscillations in the 10–20 Hz frequency range. Oscillations are highly coherent across the OB and PCX. Slight time lag of the PCX signal relative to the OB signal indicates that oscillations originate in the OB. **(B)** Frequency-amplitude representations (spectra) of the local field potential recorded simultaneously from the olfactory bulb (left panel) and piriform cortex (right panel) in a 5-day old awake rat pup. Spectra are based on 10 odor presentations. The peak around 1 Hz indicates respiration-induced modulation. Vertical dashed lines in panel **(B)** indicate peak frequency of odor-evoked activity. Figures adapted from Zhang et al. (2020).

**FIGURE 3:**
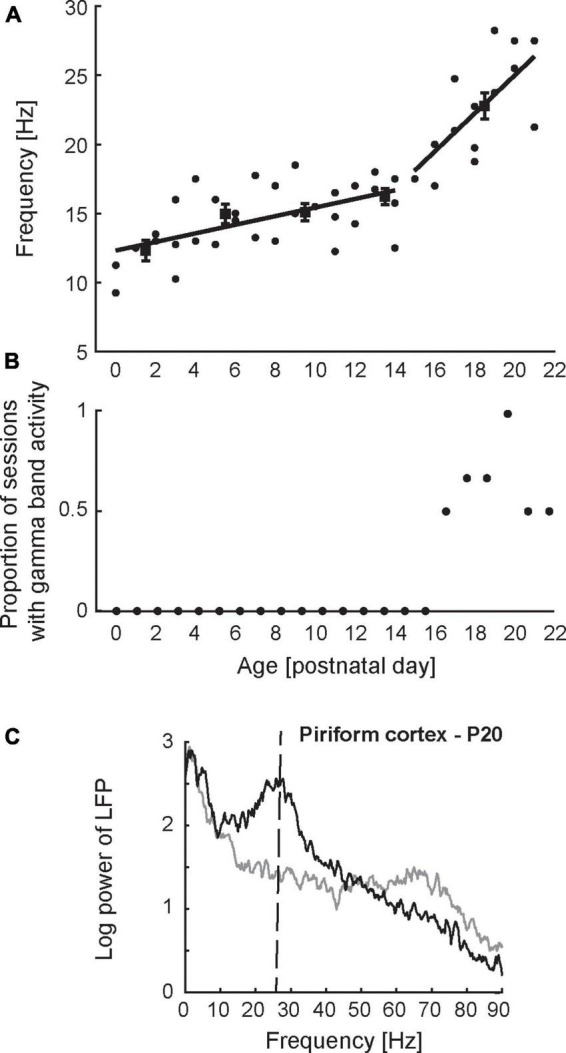
Developmental dynamics of network-level activity in the piriform cortex. **(A)** Peak frequency of odor-evoked activity in PCX as a function of age. Each datapoint represents a single recording session from a unique animal. Peak frequency was determined based on the spectrum obtained from the local field potential recorded during odor presentation (*n* = 10 trials). Lines represent the best piecewise linear fit to the data. **(B)** Proportion of recording sessions exhibiting spontaneous gamma-band oscillations (60–90 Hz) in the PCX as a function of age. The presence of gamma-band oscillations was determined based on the spectrum obtained from the local field potential recorded before stimulus presentation (*n* = 10 trials/session). Gamma band oscillations were not observed in the PCX prior to postnatal day 16. **(C)** Spectrum of local field potential activity recorded from the PCX in a 20-day old rat pup. Mature network activity in the PCX emerges after P15 and is characterized by odor-evoked beta oscillations (20–30 Hz) and spontaneous gamma oscillations (60–90 Hz). Vertical dashed line in panel **(C)** indicates peak frequency of odor-evoked activity. Figures adapted from Zhang et al. (2020).

The studies reviewed above demonstrate that the neonatal olfactory system features both mature and immature local circuit motifs that support unique neonatal olfactory function. However, it remains largely unknown how these circuits interact to process odor input in awake behaving animals. In adult rodents, processing of odor inputs in awake behaving animals is characterized by coherent oscillatory activity at multiple spatial scales across the olfactory network (Kay et al., 2009). To gain insight into how neonatal circuits function to generate dynamic neural activity patterns reflective of information processing *in vivo*, a recent study applied electrophysiological recordings in awake rat pups during the first 3 weeks of life while they actively sampled odor stimuli (Zhang et al., 2020). Figure 2A shows traces of local field potential activity recorded simultaneously from the OB and PCX at P5. Orthonasal presentation of an odor stimulus evoked bursts of 10–20 Hz oscillatory activity with each inhalation in both OB and PCX. Oscillatory activity is apparent as a distinct peak in the frequency representation of the local field potential shown in Figure 2B. Oscillations in the 10–20 Hz range were present at birth (see Figure 3A), and similar oscillations were observed in a different study that recorded from the OB of anesthetized rat pups at P7 (Fletcher et al., 2005). Neonatal oscillations are phenomenologically analogous to beta frequency (20–30 Hz) oscillations observed in the olfactory system of adult rodents (Gray and Skinner, 1988; Kay and Freeman, 1998; Martin et al., 2004, 2006; David et al., 2015; Frederick et al., 2016). Both types of oscillations are evoked by odor stimuli, locked to the respiration cycle, coherent across the OB and PCX, and entrain cortical spiking activity. However, the underlying circuit and functional significance of neonatal oscillations remain unknown.

In adult rodents, two major types of oscillations that have been implicated in odor processing are beta and gamma oscillations (Kay et al., 2009). Both oscillations are generated in the OB, but their expression is modulated by cortical feedback projections (Gray and Skinner, 1988; Martin et al., 2004, 2006; David et al., 2015). As laid out above, cortical feedback in adults targets inhibitory granule cells (Price and Powell, 1970), which form reciprocal synapses with mitral cells (Strowbridge, 2009), thereby controlling excitability. In the presence of odor input—when cortical feedback is strong—mitral cells are in a low-excitability state and the OB and PCX exhibit coherent beta oscillations. In the absence of odor stimuli—when cortical feedback is weak—mitral cells are in a high-excitability state and exhibit gamma frequency oscillations. Functionally, beta oscillations reflect long-range functional interactions between the OB and PCX necessary for olfactory learning (Martin et al., 2004). Gamma oscillations on the other hand reflect local interactions between mitral and granule cells, and aid in fine discrimination between bulbar odor representations (Beshel et al., 2007; Martin and Ravel, 2014; Frederick et al., 2016). In the neonatal olfactory system, 10–20 Hz oscillations also originate from the OB (Zhang et al., 2020). Moreover, the work reviewed above shows that the neonatal OB receives cortical feedback projections and features inhibitory mitral-granule cell synapses. This leaves open the possibility that the neonatal OB can generate oscillations that exceed the 10–20 Hz range. Regarding beta (20–30 Hz) oscillations, one possibility is that cortical feedback (and as a result: inhibition in the OB) is particularly strong in neonates, suppressing oscillations to a low (10–20 Hz) frequency range. This may be adaptive in allowing functional interactions of the relatively mature OB with a relatively immature PCX that is incapable of sustaining beta frequency oscillations. Another possibility (not mutually exclusive with the former) is that the neonatal OB can switch between long-range (10–20 Hz) and local scale (gamma) processing modes depending on context. Alternatively, some aspects of local mitral-granule cell circuitry may not yet have fully matured in neonates, preventing the expression of gamma oscillations. To date, gamma oscillations have not been observed in neonatal animals, but future work combining *in vivo* recordings and circuit-breaking techniques in awake behaving neonatal animals will test the context-dependence of neonatal oscillatory activity, and the role of cortical feedback.

A third network-level activity pattern that occurs in the olfactory system *in vivo* are oscillations in the theta frequency range (8–12 Hz). Like beta oscillations, theta oscillations reflect long-range functional interactions, and have been observed across olfactory and limbic areas such as the entorhinal cortex and hippocampus in adult rats (Kay, 2005). A recent study in neonatal mouse pups demonstrated coherent theta oscillations across the OB and entorhinal cortex (Gretenkord et al., 2019). In general, functional interactions between olfactory and limbic circuits may facilitate learning and memory. Additionally, the authors speculate that activity patterns originating from the OB may affect the maturation of downstream systems. Given the accelerated developmental profile of the OB, a relatively mature OB that continuously generates structured activity patterns in response to external input may play an instructional or permissive role in the development of connected systems. Indeed, Gretenkord et al. (2019) showed that experimentally-induced anosmia during early life led to immediate loss of coherent oscillatory activity between the OB and entorhinal cortex, and to long-term disruption of neural activity patterns within the entorhinal cortex.

## Developmental changes in olfactory circuit function

Few studies have tracked *in vivo* neural activity patterns in the olfactory system [or any other sensory system for that matter, see Colonnese et al. (2010) for an exceptional rare example] across early development, and it remains largely unknown how the system transitions from the neonatal state described above to a mature state. Major developmental milestones occur during the third week of life, including eye opening and the onset of autonomous exploratory behavior (Bolles and Woods, 1964; Welker, 1964; Zhang et al., 2020). Roughly aligned with these milestones, Zhang et al. (2020) observed profound changes in *in vivo* network-level activity pattern. Figure 3A shows peak frequency of neonatal odor-evoked oscillations as a function of age. Prior to P15, olfactory network oscillations in response to odor stimuli are remarkably stable, consisting of the 10–20 Hz oscillations described above. After P15, the frequency of odor-evoked oscillations undergoes an abrupt increase from 10–20 Hz to mature beta frequency range (20–30 Hz). Aligned with this rapid increase in odor-evoked oscillations is the appearance of mature spontaneous gamma oscillations (40–90 Hz) in the PCX (Figure 3B). Figure 3C shows the spectrum of mature network-level activity in the PCX, characterized by odor-evoked beta and spontaneous gamma oscillations. Future work will test whether the appearance of gamma oscillations in PCX occurs in parallel with the OB or whether the two brain regions follow independent developmental time courses.

Changes in network-level activity patterns are certainly the result of changes in brain structure. However, developmental changes in structure and function appear to follow divergent dynamics. Whereas network activity patterns recorded *in vivo* are stable for protracted periods of development and undergo abrupt changes, maturation at the molecular, cellular and synaptic level typically progresses continuously. In the OB, the number of glomeruli gradually increases (LaMantia and Purves, 1989), and mitral cells undergo gradual changes in biophysical properties during the first 3 weeks of life (Yu et al., 2015). The number of granule cells also gradually increases during the first 2 weeks of life (Hinds, 1968; Mair et al., 1982), paralleled by an increase in inhibitory synapse strength, and followed by a decrease in inhibition after 2 weeks (Wilson and Leon, 1986, 1987; Dietz et al., 2011). In the PCX, myelination of the lateral olfactory tract (which carries the afferent axons from mitral cells to the PCX) (Schwob et al., 1984) and anterior commissure (which connects the PCX across the two hemispheres) (Martin-Lopez et al., 2018) reaches adult levels by 2 weeks of life. Pyramidal cells continue to differentiate and undergo gradual changes in morphology during the first 3 weeks of life (Poo and Isaacson, 2007; Sarma et al., 2011; Oruro et al., 2020). The number of inhibitory neurons in the PCX decreases during the first 2 weeks, followed by an increase in the number of inhibitory synapses after 2 weeks of life (Schwob et al., 1984; Westenbroek et al., 1988; Sarma et al., 2011; Pardo et al., 2018).

It remains unclear how network-level activity remains stable during the first 2 weeks despite profound, gradual structural changes. Previous work on the developing visual system suggests that abrupt developmental changes in network-level activity patterns can result from dynamic interactions between gradually maturing circuit elements (Romagnoni et al., 2020). Abrupt changes in network-level activity patterns would ensure stable function during early developmental stages while incorporating new functionality in preparation for the next developmental stage. Indeed, developmental changes in network-level activity in the visual system occur abruptly and in a coordinated manner around the time of eye opening (Golshani et al., 2009; Rochefort et al., 2009; Colonnese et al., 2010; Colonnese, 2014; Hoy and Niell, 2015). Another unanswered question is whether abrupt developmental changes in olfactory function are driven by internal factors, or dependent on sensory experience. Relevant in this respect is the potential role inhibitory synapse maturation plays in this process. As reviewed above, both the OB and PCX undergo substantial changes in the strength of inhibitory synapses after 2 weeks of life: the OB shows a marked decrease in inhibition starting at P15; the PCX a marked increase around the same time. A role for the maturation of inhibition in shaping network-level function is supported by findings regarding the development of oscillatory activity in the PCX. The increase in inhibitory synapse density in the PCX after P15 parallels the temporal profile of rapid increases in oscillation frequency from 10–20 Hz to beta frequency (20–30 Hz), as well as the emergence of gamma oscillations (Figure 3B). Both beta and gamma oscillations in the PCX critically rely on balanced intracortical excitatory-inhibitory interactions (Poo and Isaacson, 2009; Gonzalez et al., 2023). The finding that beta and gamma oscillations in the PCX emerge simultaneously (Figures 3A, B) further suggests that maturation of inhibition may serve as a common factor driving these developmental processes in a coordinated manner. The idea that maturation of inhibition shapes developmental changes in network-level activity is also consistent with previous work in neocortical sensory systems, and some of these studies indicate a role of sensory experience in shaping the maturation of inhibition in cortical circuits (Miao et al., 2016; Guan et al., 2017; Tatti et al., 2017). Future work using sensory deprivation protocols will elucidate the role of experience in the development of information processing in the olfactory system.

## Conclusion

The research reviewed above demonstrates that the neonatal olfactory system uses unique, developmentally-transient processing mechanisms that are adapted to immature structural features, yet perform age-appropriate function. *In vitro* preparations and work in anesthetized animals continue to provide mechanistic insight into how various components of the neonatal olfactory circuit function at the molecular, cellular and synaptic level. In addition, recent efforts to studying neonatal olfactory processing using *in vivo* techniques in awake animals is starting to gain unique insight into how components of the neonatal olfactory circuit interact to generate network-level activity patterns in response to odor stimuli. We also highlight that network-level function exhibits unique developmental dynamics across age, and that network-level activity can provide insight into how the olfactory system may affect the development of extra-olfactory circuits. Future work will combine phenomenological observation of network-level activity with controlled optogenetic and pharmacological manipulations to gain mechanistic understanding of the relation between molecular, cellular, and synaptic processes and network-level function. Finally, placing *in vivo* preparations in different environmental contexts will provide a detailed account of how neural function at different levels of description supports neonatal olfactory perception and behavior.

## Author contributions

JM prepared draft of the text and figures and contributed to the ideas. ZZ edited the text, drafted figures, and contributed to the ideas. Both authors contributed to the article and approved the submitted version.
